# The Hawthorne Effect: a randomised, controlled trial

**DOI:** 10.1186/1471-2288-7-30

**Published:** 2007-07-03

**Authors:** Rob McCarney, James Warner, Steve Iliffe, Robbert van Haselen, Mark Griffin, Peter Fisher

**Affiliations:** 1 Department of Psychological Medicine, Imperial College London, UK; 2 Department of Primary Care and Population Sciences, University College London, UK; 3 International Institute for Integrated Medicine, France; 4 Royal London Homœopathic Hospital, London UK

## Abstract

**Background:**

The 'Hawthorne Effect' may be an important factor affecting the generalisability of clinical research to routine practice, but has been little studied. Hawthorne Effects have been reported in previous clinical trials in dementia but to our knowledge, no attempt has been made to quantify them. Our aim was to compare minimal follow-up to intensive follow-up in participants in a placebo controlled trial of Ginkgo biloba for treating mild-moderate dementia.

**Methods:**

Participants in a dementia trial were randomised to intensive follow-up (with comprehensive assessment visits at baseline and two, four and six months post randomisation) or minimal follow-up (with an abbreviated assessment at baseline and a full assessment at six months). Our primary outcomes were cognitive functioning (ADAS-Cog) and participant and carer-rated quality of life (QOL-AD).

**Results:**

We recruited 176 participants, mainly through general practices. The main analysis was based on Intention to treat (ITT), with available data. In the ANCOVA model with baseline score as a co-variate, follow-up group had a significant effect on outcome at six months on the ADAS-Cog score (n = 140; mean difference = -2.018; 95%CI -3.914, -0.121; p = 0.037 favouring the intensive follow-up group), and on participant-rated quality of life score (n = 142; mean difference = -1.382; 95%CI -2.642, -0.122; p = 0.032 favouring minimal follow-up group). There was no significant difference on carer quality of life.

**Conclusion:**

We found that more intensive follow-up of individuals in a placebo-controlled clinical trial of Ginkgo biloba for treating mild-moderate dementia resulted in a better outcome than minimal follow-up, as measured by their cognitive functioning.

**Trial registration:**

Current controlled trials: ISRCTN45577048

## Background

The so-called 'Hawthorne Effect' may be an important factor affecting the generalisability of clinical research to routine practice, but has been little studied. Consider the following scenario: a clinician assesses a patient with Alzheimer's disease, decides to prescribe a treatment to improve cognition and reassesses the patient six months later. At follow-up, the patient appears marginally worse, so, the clinician, mindful of the fact that in published dementia trials, people receiving active medication often appear better or stable stops the drug, believing this to be a treatment failure. But the clinician has not taken into account the possible impact of the Hawthorne Effect on the trial results that guide his practice- patients in clinical trials appear to fare better than those in routine practice by virtue of their participation. He may have stopped his patient's medication unnecessarily.

The Hawthorne Effect was first reported following an extensive research programme investigating methods of increasing productivity in the Western Electrical Company's Hawthorne Works in Chicago during the 1920s and 30s [1,2]. The finding of enduring interest was that no matter what change was introduced to working conditions, the result was increased productivity. For example, improving or reducing the lighting in the production areas under test produced similar effects. It has been defined as 'an increase in worker productivity produced by the psychological stimulus of being singled out and made to feel important' [3]. Subsequently the definition has been broadened; here it refers to treatment response rather than productivity.

Although first reported in industrial research, the Hawthorne Effect clearly may have its implications for clinical research and its generalisability to routine practice. If there is a demonstrable benefit from participating in clinical research, *for whatever reason*, then this has implications for good clinical practice and for improving care [4,5]. The Hawthorne Effect is a component of the non-specific effects of trial participation, but is not controlled for by usual controlled trial designs.

Most clinical trials are unable to quantify the magnitude of the Hawthorne Effect because its defining features, such as extra attention by researchers and higher levels of clinical surveillance, apply equally to treatment and control arms. Although the Hawthorne Effect should not affect assessment of the difference between intervention and control, it may result in an inflated estimate of effect size in routine clinical settings by over-estimating response in both groups.

Hawthorne Effects have been suggested in previous clinical trials in dementia but as far as we are able to determine, no attempt has been made to quantify them. To quantify the magnitude of the Hawthorne Effect, in the context of a placebo-controlled study of an intervention for treating mild-moderate dementia, we compared intensive to minimal follow-up in a randomised fashion. Our aim was to determine whether intensive follow-up had an effect on cognition, quality of life, behavioural functioning and/or psychopathology, as compared to minimal follow-up.

Our primary null hypothesis for this component of the trial was that compared to minimal follow-up, intensive follow-up would have no additional effect on participants' in a placebo controlled trial of Ginkgo biloba for treating mild-moderate dementia in a community setting, as measured by the ADAS-Cog (the Alzheimer's Disease Assessment Scale – Cognitive subscale) [6] and the participant's quality of life, as measured by the QOL-AD (Quality of Life in Alzheimer's Disease assessment scale) [7].

## Methods

We conducted a 2 × 2 factorial, community-based, pragmatic, randomised, double-blind, parallel-group trial in which participants were treated, double-blind with an high-purity extract of Ginkgo biloba (120 mg daily) or placebo for six months (the DIGGER trial: Dementia In General Practice Ginkgo Extract Research). In addition to randomisation to treatment, participants were randomised to intensive or minimal follow-up (see below for details). The factorial design was economical in terms of the numbers of patients required to address the two questions. The trial was approved by South West Multi-Centre Research Ethics Committee (ref:MREC/02/6/35) and was registered with Current Controlled Trials(ISRCTN45577048). The trial was sponsored by London West Mental Health Research and Development Consortium, and funded by the Alzheimer's Society. There was extensive consumer involvement, through the Alzheimer's Society, at all stages.

### Participants

We recruited participants in Greater London (UK) and adjacent regions through referrals from general practitioners, old age psychiatrists and other health care professionals; and from direct responses to advertising in Alzheimer Society newsletters, London-based newspapers, and posters in Age Concern centres.

Eligibility was determined with reference to the following:

#### Inclusion criteria

• Aged 55 years or over

• Presence of a carer able to report on the functioning of the participant

• Informed consent of the participant or in the case of an individual who lacks the capacity to give their consent, their assent and the agreement of their nominated carer

• Sufficient command of English to complete questionnaires

• Clinical diagnosis of dementia (sub-classified using DSM-IV criteria) [8]

• A Mini Mental State Examination (MMSE) [9] score of 12–26 inclusive

• Living in the community

#### Exclusion criteria

• Use of Ginkgo in two weeks prior to the baseline assessment

• Commencement of cholinesterase inhibiting drugs within 2 months of baseline or during follow-up

• Concomitant warfarin therapy

• Known bleeding abnormalities

### Interventions (follow-up)

In order to assess the Hawthorne Effect, participants were randomised to intensive follow-up (with comprehensive assessment visits at baseline and two, four and six months post randomisation) or minimal follow-up (with an abbreviated assessment at baseline and a full assessment at six months). To minimise the chances that any difference was due to medication intake, the minimal follow-up group was sent their study medication by post (Royal Mail) at two-monthly intervals. All visits were domiciliary.

### Outcomes

Our primary outcome measures were: i) cognitive functioning, as measured by the ADAS-Cog, a 0–70 point scale with a higher score indicating worse cognition; and ii) quality of life, rated by the participant and their nominated carer, as measured by the QOL-AD, both 13-item scales, scoring between 13 and 52 points with a higher score indicating better quality of life. Both outcome measures are validated tools previously (and in most cases commonly) used in dementia trials. Other measures undertaken at each assessment as part of the therapy component of the trial included a measure of psychopathology, a social behaviour scale, a caregiver burden scale and a blood test for coagulation time. We decided, *a priori*, not to consider these outcomes in the Hawthorne analysis. Each patient and caregiver assessment took approximately 1.5 to 2 hours. All outcomes were administered by a trained researcher during a home visit. The ADAS-Cog was scored by the researcher; all other measures were scored by the participant or their carer.

### Sample size

As we knew of no studies that had attempted such a comparison before, we did not power the study to detect a difference between the follow-up groups. Instead we relied on the sample size calculation for effect of Ginkgo over placebo, which gave a target sample size of 200 participants. This sample size was sufficient to detect a 4-point between-group difference in ADAS-cog with 80% power at 5% significance.

### Randomisation procedure and blinding

A 2 × 2 factorial design involving two separate randomisations, resulting in participants being randomised to one of four arms, was employed. Both factors consisted of two levels: medication group (Ginkgo and placebo); and level of follow-up (minimal or intensive). This produced four groups: the Ginkgo group with intensive follow-up, the Ginkgo group with minimal follow-up, the placebo group with intensive follow-up and the placebo group with minimal follow-up. The randomisation codes were generated usingthe computer algorithm RCODE v.4.8 (Schwabe, 2002).

The Ginkgo vs. placebo component of the trial was double-blind, but it was not possible to blind the Hawthorne component of the trial as both researchers and participants needed to know when the next assessment would be.

### Statistical methods

The primary analysis was intention to treat (ITT) with available data, by randomisation group. We were interested in the effects of intensity of follow-up; participants who withdrew from the study were not exposed to the "intervention", so analysis with imputed data was not thought appropriate.

In order to take account of the baseline score, when comparing the outcomes between the treatment groups, analysis of co-variance (ANCOVA) was used. Normal distributions were assumed for the ANCOVA analyses and were checked using residuals from the regression models. If data showed substantial deviations from these assumptions, appropriate transformations were applied. Adjusted differences in means (β) are presented with 95% confidence intervals and p values. Chi-square test was used for comparison of proportions. The analysis was conducted using SPSS v.13 (SPSS Corporation, 2004) and STATAv.8.1 (STATA Corporation, 2003).

## Results

**Figure 1:**
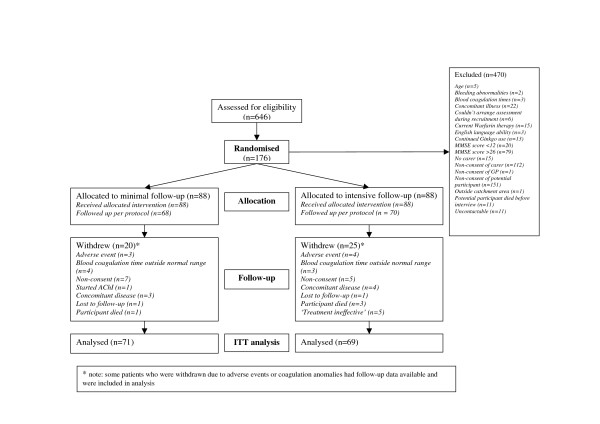
Participant flowchart.

Figure 1 details participant flow through the trial for the Hawthorne analysis. Recruitment took place between February 2003 and June 2005. Table 1 presents baseline demographic and clinical characteristics of the participants and baseline demographic characteristics of their carers. 119 GP practices, representing 388 individual GPs, recruited for the trial. Of the 176 participants in the trial, 132 (75%) were recruited through their GP.

**Table 1:** Baseline demographic and clinical characteristics of participants and demographic characteristics of carers, by follow-up group

**PARTICIPANTS**			
**Characteristic**	**Minimal group** (n = 88)	**Intensive group** (n = 88)	**Total sample** (n = 176)

Mean age*	79.2 (6.8)	79.7 (8.4)	79.5 (7.6)
Females, Males††	55, 33 (62.5%)	52, 36 (59.1%)	107, 69 (60.8%)
Ethnicity††	White: 83 (94.3%) Mixed: 1 (1.1%) Indian: 2 (2.3%) Black: 2 (2.3%)	White: 84 (95.5%) Indian: 1 (1.1%) Black: 3 (3.4%)	White: 167 (94.9%) Mixed: 1 (0.6%) Asian or Asian British: 3 (1.7%) Black or Black British: 3 (3.4%)
Median years of education†	10.0 (9.0, 14.0)	10.0 (9.0, 13.1)	10.0 (9.0, 13.3)
Number with Alzheimer's disease, vascular dementia diagnosis††	71, 17 (80.7%)	77, 11 (87.5%)	148, 28 (84.1%)
Evidence of vascular pathology††	40 (45.5%)	46 (52.3%)	86 (48.9%)
Median MMSE score†	22.0 (15.0, 26.0)	23.0 (13.9, 26.0)	22.0 (15.0, 26.0)
Mean ADAS-Cog score*	23.2 (10.2)	22.3 (8.9)	22.7 (9.6)
Median carer-reported duration of dementia in years†	3.0 (1.0, 8.1)	3.0 (1.3, 8.0)	3.0 (1.0, 8.0)
AChI use††	28 (31.8%)	30 (34.1%)	58 (33.0%)
			

**CARERS**§			

**Characteristic**	**Minimal group** (n = 88)	**Intensive group** (n = 88)	**Total sample** (n = 176)

Mean age*	63.1 (14.4)	65.2 (12.9)	64.1 (13.7)
Females, Males††	77, 11 (87.5%)	75, 13 (85.2%)	152, 24 (86.4%)
Number who are the partner of the participant††	45 (51.1%)	48 (54.5%)	93 (52.8%)
Number who live-in††	56 (63.6%)	59 (67.0%)	115 (65.3%)
Number who are the informal (unpaid) carer††	83 (94.3%)	86 (97.7%)	169 (96.0%)

* Mean scores are reported with standard deviations.

† Median scores are reported with 10^th ^& 90^th ^percentiles.

†† Numbers reported with percentage of group (e.g. Ginkgo group); or percentage of females or AD sufferers respectively.

§ Two participants study carers changed during follow-up. Data is presented for the carer at the end of follow-up.

At baseline and prior to randomisation, participants and their carers were asked which follow-up group they would prefer, and told that their preference would not affect the group to which they were assigned (Table 2). There was no interaction between the two randomisation factors (intensity of follow-up and treatment group).

**Table 2:** Participant and carer follow-up group preference, as stated prior to randomisation*

**Participants**			
	**Minimal group** (n = 88)	**Intensive group** (n = 88)	**Total sample** (n = 176)

Prefer minimal	10 (5.8%)	9 (5.3%)	19 (11.1%)
Prefer intensive	17 (9.9%)	19 (11.1%)	36 (21.1%)
No preference	59 (34.5%)	57 (33.3%)	116 (67.8%)
Missing	2 (0.02%)	3 (0.03%)	5 (0.06%)
			

**Carers**			

Prefer minimal	9 (5.1%)	9 (5.1%)	18 (10.2%)
Prefer intensive	35 (19.9%)	30 (17.0%)	65 (36.9%)
No preference	44 (25.0%)	49 (27.8%)	93 (52.8%)

* Percentages given are of total number of participants or total number of carers respectively.

Data on effectiveness of Ginkgo versus placebo, and adverse events will be reported elsewhere. To summarise these findings however, in the ANCOVA model with baseline score as a co-variate (n = 176), we found no evidence that a standard dose of high purity Ginkgo biloba confers benefit over placebo in mild-moderate dementia over six months.

### Main analysis

The main analysis for this paper was based on Intention to treat (ITT), with available data. In the ANCOVA model with baseline score as a co-variate, follow-up group had a significant effect on outcome at six months on the ADAS-Cog score (n = 140; β = 2.018; 95%CI -3.914, -0.121; p = 0.037), favouring the intensive follow-up group. There was also a significant effect on participant-rated quality of life score (n = 142; β = -1.382; 95%CI -2.642, -0.122; p = 0.032) favouring the minimal follow-up group. There was no significant effect of follow-up group on carer-rated quality of life score (n = 131; β = 0.169; 95%CI -1.489, 1.827; p = 0.841).

### Per protocol analysis

This included individuals who were followed-up at all allocated time-points; who provided data at baseline and six months; and who did not withdraw. In the ANCOVA model with baseline score as a co-variate, follow-up group had a significant effect on outcome at six months on the ADAS-Cog score (n = 116; β = -2.376; 95%CI -4.519, -0.233; p = 0.030), which favoured the intensive follow-up group. Follow-up group did not have a significant effect on neither participant-rated quality of life score (n = 115; β = -1.238; 95%CI -2.683, 0.207; p = 0.092) or carer-rated quality of life score (n = 106; β = 0.317; 95%CI -1.359, 1.994; p = 0.708).

The main analysis is based on evaluable data with 71 participants in the minimal follow-up group and 69 in the intensive follow-up group. Twelve participants (13.7%) in the minimal followup group and 26 (29.5%) in the intensive follow-up group were not followed-up according to protocol (i.e. two or four visits respectively in the six months). This difference is statistically significant (χ = 6.578; d.f. = 1; p = 0.010). The rate of withdrawal was similar between groups (22 in the minimal group and 23 in the intensive group) as was compliance with trial medication (taking 80% or more of the allocation – 72 in the minimal follow-up group and 69 in the intensive follow-up group). Neither of these differences is significant. There was no significant difference in the number of GP contacts during the study between intensive (16 contacts) and minimal groups (16 contacts) evaluated by self-report at 6 months.

## Discussion

We found that more intensive follow-up of individuals in a placebo-controlled clinical trial of Ginkgo biloba for treating mild-moderate dementia resulted in a better outcome than minimal follow-up, as measured by their cognitive functioning. Participants in our study had a maximum of four assessment points at 2 monthly intervals: less intensive than some other trials. For example participants in two trials of donepezil had 7 assessments in 14 weeks and 26 weeks respectively [10,11], Such assessment protocols may result in a greater Hawthorne Effect. This is the first example of a randomised controlled investigation of trial effects where both groups have comparable (baseline) characteristics. Our findings accord with previous literature [4,5] – which suggest that there *may *be a small, positive effect of trial participation on response – and our results suggests that the intensity of follow-up could be a factor here.

The mean difference between the follow-up groups was two points on the ADAS-Cog. This may not be regarded as particularly clinically significant, but is of similar magnitude to that reported in randomised controlled trials of cholinesterase inhibitors for dementia [12].

We also found a significant difference in participant-rated quality of life score in the main analysis, suggesting quality of life was worse in participants who were intensively followed-up. This may seem to be contrary to the impact on cognition, as improved quality of life may be expected to mirror better cognition scores. However, improved ADAS-cog scores in this study may not reflect better cognition (participation is a study *per se *is unlikely to improve cognitive performance), but be due to Hawthorne or learning effects. Evidence of a possible Hawthorne effect may be supported by the finding that more intensive contact leads to lower reported quality of life. Previous literature [13] suggests that more intensive contact may lead to a better recognition of needs. This may in part be due to more 'honest' reporting of needs if intensively followed-up as there is a better relationship with the care provider. A similar honesty premium may have operated in the intensively followed up group here. Another possibility is that more intensive contact with study personnel led to greater awareness of the diagnosis and resultant disability, impairing perception of quality of life. This explanation may account for the lack of differences in carer QOL.

Some researchers have criticised the interpretation of the original research about the Hawthorne Effect [14]. Other possible variables which may have contributed to the observed effect include managerial input; the fear of losing one's job during The Great Depression; the duration of rest breaks; and the changing of participants and human relations, among many factors contributing to the overall lack of scientific rigour. Indeed the first statistical analysis of the findings found most of the variation was explained by other factors [3]; other researchers argue that the effect was minimal at best [15,16]; and yet others have not managed to replicate the findings [17]. As a result the usefulness of the term has been questioned; as it is effectively a catch-all term for labelling psychological and social factors that weren't controlled for or that cannot be explained. Parsons [18] defined it as 'the confounding that occurs if experimenters fail to realize how the consequences of subjects' performance affect what subjects do". Nevertheless and perhaps erroneously, the term 'Hawthorne Effect', coined by French [19] many years after the original experiments, has come to be understood as, the effect on outcome through the participation in research.

There are limitations to our study. We have looked at a specific group: individuals with mild-moderate dementia in a community setting, and the effect may be limited to this group especially as learning, and/or habituation to the assessment process over time, may be significant components of improved outcome in dementia trials. A Hawthorne Effect has been previously suggested in dementia trials [20,21], which drove our investigation. It has been suggested [20,21] that an observed effect amongst such a group may be due to a learning effect whereby familiarity with the instruments results in better performance. To minimise learning effects, we used different word lists for the memory items (sections one and seven of the ADAS-Cog) at each follow-up point. These items account for the majority of variation in ADAS-Cog scores amongst individuals with mild-moderate dementia [22]. Furthermore, the overall test-retest coefficient was found to be 0.91 over a six week period [23], which does not suggest there is a significant learning effect associated with the ADASCog. The opposite argument can also be made: people with dementia have a reduced ability to learn and learning effects may thus be smaller in this group than others. Our results do not allow us to rule out either possibility, and if this is the case, the Hawthorne effect may be larger in non-dementia trials.

Another limitation is the lack of blinding of participants and researchers to follow-up group. This may have a 'nocebo effect' in that those minimally followed-up may feel that they are not getting the 'best care' during their participation. However as Table 2 shows, two-thirds of the participants and over half of the carers expressed no preference in respect of follow-up frequency. Furthermore, the distribution of preference was similar between the groups (i.e. half of those participants who preferred minimal follow-up received minimal follow-up). This suggests that such an effect may not be of great import. A further possible limitation is that we assessed participants in their own home, rather than in hospital as most studies do, and this may have reduced the impact of trial participation.

The situation around adherence to protocol is complex. There is an imbalance in the follow-up rates between the groups: intensive follow up patients were less likely to be followed up according to the protocol. This may be due to the greater demands on participants and their carers in this group. This may introduce bias, with more compliant participants remaining in the study. However the main analysis is based on evaluable data, with similar numbers in the groups providing data at six months (71 in the minimal, 69 in the intensive). The imbalance in numbers followed-up according to protocol is due to missed two or four month assessments in the intensive follow-up group, making their assessment schedule more similar to that of the minimal follow up group. This reduces the difference between groups and is likely to underestimate the effect. Rates of attrition and compliance to the trial medication did not differ significantly between groups.

It is possible that there is a threshold effect: any participation in a clinical trial is associated with a different outcome from that of routine care, but the difference between levels of follow up within a clinical trial is small. This is perhaps the most relevant question in terms of generalisability of clinical trial findings to routine practice, but we did not attempt to answer it, and a different methodology would have been required to do so.

We draw two main implications from our findings. The first relates to how treatment effects may be over-estimated as a result of follow-up in trials. If there is an uneven rate of drop-out between groups, the benefit to the group with less attrition may be inflated because of the added effect of follow-up. In dementia research, more people tend to drop-out of the treatment arm; this would lead to an underestimate of the effect size of the intervention. Secondly it may have implications for best practice because if trial participation improves outcome (for whatever reason), it can be argued that routine treatment should be carried out under similar conditions to clinical trials [5]. This, of course, may have resource implications and the issue might be best considered in the context of maximising the non-specific effects of treatment. If our findings are replicated, the presence of a Hawthorne Effect should be considered when interpreting the results of treatment trials in dementia.

Hrobjartsson and Gotzsche [24] in a systematic review of placebo effects found placebo had minimal clinical effect compared to usual treatment. Braunholtz et al [4] systematically reviewed whether participation in clinical trials is beneficial or otherwise to patients, they defined separately treatment, protocol, care, Hawthorne and placebo effects, but remarked that it is difficult to tease such effects apart. The conclusion was that there is weak and inconclusive evidence of benefit of a Hawthorne Effect. A 'structured' review [5] compared outcomes in cancer patients treated within and outside clinical trials and found little evidence in support of a beneficial trial effect. Both these reviews focussed on cancer and both groups of authors agreed on the paucity of good quality evidence, considering the reason for this to be the methodological difficulties inherent in trying to make such evaluations in an ethical manner, with full and informed consent while maintaining comparable trial and non-trial groups.

## Conclusion

In a randomised, non-blind comparison of intensive and minimal follow up of community dwelling patients suffering from mild-to-moderate dementia, we found evidence of a small 'Hawthorne Effect'. This may be due to effects other than being observed, such as learning effects of repeated exposure to the ADAS cog or greater familiarity with the research process. In any event, a non-treatment driven effect remains intriguing and clinically relevant, whatever the cause.

## Competing interests

RM, JW, SI, MG and PF have no possible conflicts of interest to declare. RvH provided some consultancy services to Schwabe Pharma in 2005, unrelated to this research.

## Authors' contributions

RM, JW, SI, RvH and PF participated in the development, implementation and management of this project and were involved in drafting the manuscript. MG participated in the statistical aspects of the design and analysis and in drafting this part of the manuscript.

## Pre-publication history

The pre-publication history for this paper can be accessed here:



## References

[B1] Mayo E (1993). The human problems of an industrial civilization.

[B2] Roethlisberger FJ, Dickson WJ (1939). Management and the Worker.

[B3] Franke RH, Kaul JD (1978). The Hawthorne experiments: First statistical interpretation. Am Sociol Rev.

[B4] Braunholtz DA, Edwards SJ, Lilford RJ (2001). Are randomized clinical trials good for us (in the short term)? Evidence for a "trial effect". [Review] [26 refs]. Journal of Clinical Epidemiology.

[B5] Peppercorn JM, Weeks JC, Cook EF, Joffe S (2004). Comparison of outcomes in cancer patients treated within and outside clinical trials: conceptual framework and structured review.[see comment]. [Review] [52 refs]. Lancet.

[B6] Rosen WG, Mohs RC, Davis KL (1984). A new rating scale for Alzheimer's disease. American Journal of Psychiatry.

[B7] Logsdon RC, Gibbons LE, McCurry SM, al. (1999). Quality of life in Alzheimer's disease: patient and caregiver reports. Journal of Mental Health and Ageing.

[B8] American Psychiatric Association (2000). Diagnostic and Statistical Manual of Mental Disorders DSM-IV-TR (Text Revision).

[B9] Folstein MF, Folstein SE, McHugh PR (1975). 'Mini-mental state': a practical method for grading the cognitive state of patients for the clinician. Journal of Psychiatric Research.

[B10] Rogers SL, Friedhoff LT (1996). The efficacy and safety of donepezil in patients with Alzheimer's disease: results of a US Multicentre, Randomized, Double-Blind, Placebo-Controlled Trial. The Donepezil Study Group. Dementia.

[B11] Tariot PN, Cummings JL, Katz IR, Mintzer J, Perdomo CA, Schwam EM, Whalen E (2001). A randomized, double-blind, placebo-controlled study of the efficacy and safety of donepezil in patients with Alzheimer's disease in the nursing home setting.[see comment]. Journal of the American Geriatrics Society.

[B12] Birks J (2006). Cholinesterase inhibitors for Alzheimer's disease. [Review] [47 refs]. Cochrane Database of Systematic Reviews.

[B13] O'Connor DW, Pollitt PA, Brook CP, Reiss BB, Roth M (1991). Does early intervention reduce the number of elderly people with dementia admitted to institutions for long term care?. BMJ.

[B14] Wickstrom G, Bendix T (2000). The "Hawthorne effect"--what did the original Hawthorne studies actually show?. Scandinavian Journal of Work, Environment & Health.

[B15] Rossi P, Freeman H (1989). Evaluation: a systematic approach.

[B16] Jones S (1992). Was there a Hawthorne effect?. Am J Sociol.

[B17] Rosen NA, Sales SM (1966). Behavior in a nonexperiment: the effects of behavioral field research on the work performance of factory employees. J Appl Psychol.

[B18] Parsons HM (1978). What caused the Hawthorne effect? A scientific detective story. Adm Soc.

[B19] French J, Festinger L and Katz D (1953). Experiments in field settings. Research methods in behavioural sciences.

[B20] Rogers SL, Farlow MR, Doody RS, Mohs R, Friedhoff LT, Albala B, Baumel B, Booker G, Dexter J, Farmer M, Feighner JP, Ferris S, Gordon B, Gorman DG, Hanna G, Harrell LE, Hubbard R, Kennedy J, kinney FC, McCarthy J, Scharre DW, Schaerf F, Schneider L, Seltzer B, Siegal A, Stark SR, Strauss A, Walshe TM (1998). A 24-week, double-blind, placebo-controlled trial of donepezil in patients with Alzheimer's disease. Neurology.

[B21] Rosler M, Anand R, Cicin-Sain A, Gauthier S, Agid Y, Dal Bianco P, Stahelin HB, Hartman R, Gharabawi M, Bayer T, Berger A (1999). Efficacy and safety of rivastigmine in patients with Alzheimer's disease: International randomised controlled trial. British Medical Journal.

[B22] Zec RF, Landreth ES, Vicari SK, Belman J, Feldman E, Andrise A, Robbs R, Becker R, Kumar V (1992). Alzheimer Disease Assessment Scale: a subtest analysis. Alzheimer Disease & Associated Disorders.

[B23] Talwalker S, Overall JE, Srirama MK, Gracon SI (1996). Cardinal features of cognitive dysfunction in Alzheimer's disease: a factor-analytic study of the Alzheimer's Disease Assessment Scale. Journal of Geriatric Psychiatry & Neurology.

[B24] Hrobjartsson A, Gotzsche PC (2001). Is the Placebo Powerless? — An Analysis of Clinical Trials Comparing Placebo with No Treatment. NEJM.

